# Cement Filling

**Published:** 1894-03

**Authors:** R. B. Tuller

**Affiliations:** Chicago.


ARTICLE VI.

### CEMENT FILLING.

BY DR. R. B. TULLER, CHICAGO.

I notice in September *Items* several items favoring cement fillings for permanent work. American dentists who have not been in Europe have little idea how much cement is used over there. Europeans, as a rule, will not tolerate "jewelry" in their teeth. They would rather return often for renewal of cement than be conspicuous by the glitter of gold in the mouth. Americans are frequently recognized as Americans by the display of gold in front teeth. I think the better class of dentists in this country recognize that it is bad taste to display gold fillings, and avoid it as much as possible; and people of good taste desire to avoid it. But cement is generally considered cheap dentistry and not permanent. It is not easy to command a proper fee for skilful and artistic work, simply because it is only cement. I have a filling of cement in a lower molar which has done service for thirteen years, and is only defective now in being somewhat worn away. But I must confess I cannot make cement fillings do such service. Perhaps I could if I tried, and could get proper pay for it. There are many cases where it would be desirable, in many ways, if only permanent. In many large cavities I use it in preference to pounding in gold from the bottom up, finishing, however, with gold. I do it often to save my patient discomfort and misery, as well as for the reason that I think it means a very perfect filling. But I think most of the cement fillings should have a film of protecting substance between them and the tooth, over the region of pulp chamber. In advocating cement I think this should be kept in mind. Another thing is in regard to its adhesive quality. It does indeed cling closely to the walls of the tooth, but if any one will let a little harden on the mixing slab, or even on the rough surface of a corundum stone, and then see how easily it is dislodged after moistening, they will see that its sticking qualities depend greatly on our ability to keep moisture from between it and the walls. This would indicate the making of a cavity that has a retaining shape to a variable extent, and would also indicate the use of hot paraffin or something of that nature as a finishing coating to such fillings. I believe cement fillings could be made much more durable and satisfactory if we gave them the same study and thought we do our gold and amalgam, with a view to greater perfection.

In finishing cement with gold I generally take or make a hollow cylinder of gold, pressing together one end, making, in fact, a cup. This pressed into the cement while yet soft, prevents the moist cement from coming into the center, and the hollow is just what you want for the next piece of gold when the cement is hard enough to add it.—*Items of Interest.*

